# Calcium alginate microencapsulation of ovarian follicles impacts FSH delivery and follicle morphology

**DOI:** 10.1186/1477-7827-3-47

**Published:** 2005-09-14

**Authors:** Matthew Heise, Richard Koepsel, Alan J Russell, Elizabeth A McGee

**Affiliations:** 1 McGowan Institute for Regenerative Medicine, University of Pittsburgh, 100 Technology Dr. Suite 200, Pittsburgh, PA 15219, USA; 2 Magee-Womens Research Institute, 204 Craft Ave, Pittsburgh PA, 15213, USA; 3 Department of Obstetrics, Gynecology and Reproductive Medicine, University of Pittsburgh, Pittsburgh PA 15213, USA

## Abstract

**Background:**

We have previously shown that suspension culture prevents follicle flattening and maintains three-dimensional follicle architecture better than culture on flat plates. However, many of the follicles cultured in suspension do eventually rupture, as basement membrane integrity is lost and the three-dimensional structure of the follicle is altered. Therefore, the objective of this study is to support three-dimensional follicle architecture during in vitro growth of ovarian follicles through encapsulation in calcium alginate, while maintaining responsiveness to FSH stimulation.

**Methods:**

Preantral follicles (150 – 160 micrometers in diameter) were isolated from the ovaries of juvenile rats and grown in culture tubes or encapsulated in calcium alginate and grown in culture tubes. Previous studies revealed that follicles maintained structural integrity but did not grow as well when encapsulated in calcium alginate. In these studies, we evaluated the effect of calcium alginate on FSH-stimulated follicle growth, survival, and morphology in suspension culture. Follicles were grown under 5 culture conditions: 1) not encapsulated; with FSH in the medium, 2) encapsulated in the absence of FSH, grown in medium without FSH, 3) encapsulated with calcium alginate containing FSH but grown in medium without FSH, 4) encapsulated without FSH but grown in medium containing FSH and 5) encapsulated with calcium alginate containing FSH and in medium containing FSH. To assess growth rates, follicles were cultured for 72 hours and analyzed for follicle size increase and DNA content. Survival analysis for encapsulated and unencapsulated follicles was performed by constructing a Kaplan Meier survival curve of daily observations of intact follicle survival. Three-dimensional architecture was assessed histologically and by analysis of the pattern of connexin 43 expression in the cultured follicles.

**Results:**

In the absence of FSH, follicle diameter increased by only 6.4%. When FSH was included in the alginate bead alone or the media alone, the follicle diameter increased by 13.5% and 19.9% respectively. This was greater than follicles cultured in the absence of FSH (p < 0.05), but less than that of the FSH-treated unencapsulated follicles (p < 0.05). However, when follicles were cultured with FSH included in both the media and the bead, a 32.6% increase in follicle diameter was observed, statistically no different than the growth rate of the unencapsulated follicles grown with FSH.

**Conclusion:**

Microencapsulation supports three-dimensional follicle growth, but may limit access to hormones in the medium resulting in altered development compared to unencapsulated follicles. Inclusion of FSH in the alginate bead restores the follicle growth response to FSH, while also providing a scaffold of support for three-dimensional growth. The application of tissue engineering principles to the problems of follicle culture in vitro may provide advances applicable to fertility preservation in women and endangered species.

## Background

The culture of intact ovarian follicles is useful for the study of the regulation of folliculogenesis, but may also provide an alternative to ovarian transplant for the preservation of fertility [1]. Classically, ovarian follicle culture has been performed in culture dishes, on a flat surface. Although ovarian follicles from the mouse can readily be grown on culture plates [2,3], the maturation of follicles from larger mammals has proven to be much more difficult [1]. One cause of difficulty may be that larger follicles have different structural needs. Mouse follicles can ovulate at 400 μm in diameter [4], whereas rat preovulatory follicles are often greater than 800 μm in diameter [5]. Though this represents only a doubling of diameter, the *volume *of the rat preovulatory follicle is at least 8 times that of the mouse. The application of standard tissue engineering principles to the growth of ovarian follicles *in vitro *may provide additional tools necessary to overcome the structural challenges presented by follicles from larger mammals and make significant advances in this field.

It has been shown that suspension culture both enhances cell proliferation [6] and prevents follicle flattening and maintains three-dimensional follicle architecture better than culture on flat plates [7]. Though mouse follicles grown in a hanging drop system grew very well compared to follicles grown on flat plates, the follicle diameter was still well under 500 after 6 days of culture [6]. Rat follicles often rupture at 200 to 250 μm on flat plates. However in suspension culture follicles routinely maintain intact survival to about 400 μm. Past this size, basement membrane integrity is lost and the three-dimensional structure of the follicle is altered. One tissue engineering approach to this problem is to encapsulate the follicles. Microencapsulation has been used to provide structural support for a variety of tissues such as pancreatic islets [8] and thyroid follicles [9].

Alginate is one of the most commonly applied biomaterials for microencapsulation due to its biocompatibility, high affinity to water, and ability to form gels under mild conditions when in the presence of calcium ions [10-14]. Alginate is comprised of chains of alternating blocks of mannuronic acid, which contributes the elastic property of the gel; and guluronic acid, which contributes mechanical strength, stability, porosity, and gel forming properties [15,16]. Alginates are extracted from all species of brown algae and contain differing compositions of mannuronic acid/mannuronic acid, mannuronic acid/glucoronic acid, and glucoronic acid/glucoronic acid blocks offering a variation in strength and stability. Alginate gel beads are reported to have a high porosity range and only limit the diffusion of large proteins [12]. It has been reported that substrates of molecular weight less than 2 × 10^4^, such as glucose, L-tryptophan (MW = 204), and α-lactoalbumin (MW = 1.54 × 10^4^), are able to diffuse freely into and from calcium alginate beads at approximately the same diffusion rate as in water, while larger proteins, such as albumin (MW = 6.9 × 10^4^), could not diffuse freely into the calcium alginate beads [17]. Although there is resistance for larger proteins (MW > 2 × 10^4^) diffusing into these beads, diffusion from the bead into a surrounding solution devoid of the substrate is not hindered until the molecular weight of the substrate approaches 3 × 10^5 ^[11]. FSH has a molecular weight of 3 × 10^4 ^[18]. Thus, FSH is in the size range where hindrance may play a role in FSH availability to follicles encapsulated in calcium alginate beads.

In this study, the role of a calcium alginate scaffold on follicle growth, survival, and morphology in suspension culture was explored. Our previous studies suggested that alginate encapsulation slowed follicle growth [7]. Therefore, in these studies we have determined the growth rates of follicles in response to different delivery methods of FSH to encapsulated follicles. As a result, we have established the ability of calcium alginate to support three-dimensional follicular growth of intact follicles, but discovered that encapsulation may limit follicular access to FSH unless it is included in the alginate bead itself.

## Materials and methods

### Animals and Ovarian Dissection

All animal experiments were performed in accordance with National Institutes of Health guidelines and with institutional approval. Sprague-Dawley rats were obtained from Hilltop Lab Animals (Pittsburgh, PA) and housed under standard conditions. The animals were sacrificed by CO_2 _exposure and cervical dislocation. Ovaries were carefully dissected and placed immediately in warmed culture medium, consisting of Leibovitz L-15 Medium (Gibco BRL) with 1% bovine serum albumin (Sigma). The follicles were then mechanically dissected from the ovary as previously described [19]. All follicles used in the experiments were measured in two dimensions, using an inverted microscope fitted with an ocular micrometer. Only intact follicles that were between 150 and 160 microns in diameter were used in culture. There was no statistical difference in the starting follicle diameter of any of the groups.

### Calcium Alginate Microencapsulation

**Figure 1:**
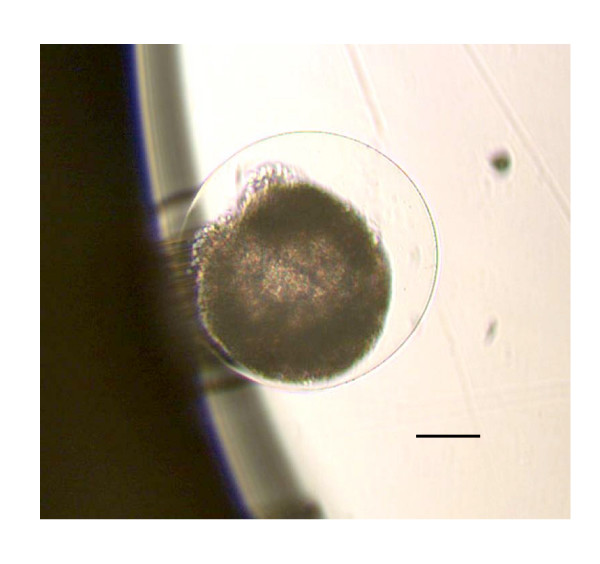
Ovarian Follicle Encapsulated in a Calcium Alginate Bead. Scale bar represents 50 μm.

After dissection, 20 to 30 follicles per group were transferred with glass pipettes to a solution of sodium alginate (1% w/v; Sigma) in distilled water. (For the groups that required FSH in the bead, follicles were transferred to a solution of sodium alginate containing recombinant Follicle Stimulating Hormone, rFSH, (Serono Laboratories, Geneva) at a concentration of 1 iu/mL.) The mixture of follicles in sodium alginate was slowly released through a 25-gauge needle as droplets falling into a beaker containing a stirred solution of CaCl_2 _(0.1 M). The droplets immediately gelled to form beads. A stream of 0.2 μm-filtered air was positioned at the tip of the needle to cut the mixture stream into small droplets to obtain beads with diameters between 250 μm to 400 μm. Beads containing individual follicles (Figure 1) were then removed from the beaker using glass pipettes and immediately transferred to media in 12 × 75 mm polypropylene culture tubes. Follicles were cultured one per tube.

### Follicle Culture

Culture media consisted of α-Minimal Essential Medium (Gibco BRL, Invitrogen Corporation, Grand Island, NY) with additives of 8-bromo-cGMP (5 mM), ITS+ (1 % solution of insulin, 10 mg/L; transferrin, 5.5 mg/L; linoleic acid, 4.7 mg/L; selenium, 5 mg/L), Pen/Strep (1 %, penicillin 100 U/ml, streptomycin 100 μg/ml), all from Sigma Chemical Co. (St. Louis, MO), and recombinant Follicle Stimulating Hormone, rFSH, (1 iu/ml; Serono Laboratories, Geneva). In the basal or negative control group, FSH was not included. Culture media was placed into 12 × 75 mm polypropylene culture test tubes (500 μl/tube) and cultured in 5 % CO_2 _and 37°C humidified incubator.

Suspension was attained by placing the 12 × 75 mm polypropylene culture tubes in a circular rotator plate (Glas-Col, Terre Haute, IN), having a diameter of 30.5 cm, which was rotated around its horizontal axis at rate between 8–15 rpm. Therefore, as the plate rotates, the tubes slowly orbit the axis of the plate and the follicle is maintained is fluid suspension. After 72 hours in culture, follicle diameter was measured once again and follicles were collected for DNA quantification and histological analysis. For survival analysis, follicles were observed daily for basement membrane integrity and signs of atresia. Atresia is readily apparent as darkening of the follicle appearance under the dissecting microscope [7,20]. Survival analysis cultures were continued for 7 days.

### DNA Quantification

To verify that increased follicle size represented increased follicle cell number, DNA quantification was performed on the cultured follicles as previously described [7]. At 72 hours of culture, the follicles were released from the beads by allowing the capsules to dissolve in sterile PBS (pH 7.4). Follicles (n = 5 for each group) were then trypsinized and DNA was extracted from each individual follicle and quantified by using the fluorescent dye, Hoechst 33258 (bisbenzimidazole; Sigma), and a microplate fluorescence reader (Perkin Elmer Life Sciences, Boston, MA) at 365 nm excitation and 450 nm emission wavelengths [21]. A range of known dilutions of salmon testes DNA (Sigma) was used to plot a standard curve from which follicle DNA content was extrapolated as previously described [7]. This experiment was repeated twice with the same result.

### Histology and Immunohistochemistry

To confirm that cultured follicles encapsulated in calcium alginate retain normal anatomic relationships between the cell types within a follicle, immunohistochemistry for connexin 43 was performed on follicles that were cultured in calcium alginate beads with and without FSH. Some additional sections were also stained with hematoxylin and eosin. At 72 hours of culture, the follicles were released from the beads by allowing the capsules to dissolve in sterile PBS (pH 7.4). Follicles were embedded in OCT for fluorescent immunostaining.

Frozen sections were cut at 5 μm and air-dried overnight at room temperature. Sections from at least 10 follicles per group were evaluated for connexin staining pattern. After a 10 minute acetone fixation, slides were again allowed to air dry, and then were rehydrated in PBS for 10 minutes. Immunohistochemistry was performed using the M.O.M. immunodetection system (Vector Laboratories, Burlingame, Calif.). All reagents were prepared according to kit directions and all steps were carried out at room temperature. Slides were incubated with blocking reagent for 1 hour. Mouse anticonnexin (Chemicon International, Temecula, CA) was prepared in diluent to a final concentration of 0.1 μg/mL and applied to the sections for 1 hour in a humidified chamber. For negative control sections, mouse IgG was substituted for the anti-connexin antibody. After a PBS wash, sections were incubated with biotinylated anti-mouse IgG for 10 minutes. Slides were again washed for 10 minutes in PBS, and then incubated with fluorescein avidin DCS (1:62.5 in PBS) for 5 minutes. After a final 10 minute PBS wash, slides were mounted with Vectashield mounting medium (Vector Laboratories) and digitally imaged using a Leica CMR fluorescence microscope.

### Statistical analysis

Statistical analysis for DNA quantification and follicle diameters was performed by analysis of variance (ANOVA) with post hoc testing. Survival data was analyzed with the Kaplan Meier program contained in the Sigma Stat 3.0 software package (Chicago, Il). Probability values less than 0.05 were used to determine significance.

## Results

### Effect of calcium alginate encapsulation and FSH treatments on follicle diameter

**Figure 2:**
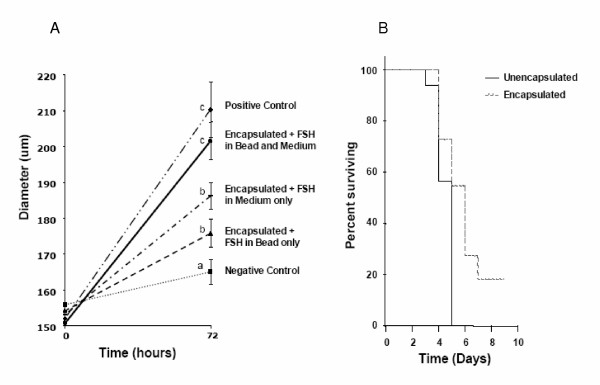
(A) Diameter of follicles cultured under different conditions over 72 h. Each line represents measured follicle diameter before and after 72 h of culture (n = 20 – 30 follicles per group). Data points are average diameter with standard error bars included for each treatment. Treatment conditions are listed to the right of the data point representing average diameter at 72 hours for each group. a, b, c represent statistically different groups (p < 0.05). (B) Kaplan Meier Survival Plot. Mean survival time for unencapsulated follicles is 4.5 days while mean survival time for encapsulated follicles is 6 days (p < 0.05).

Average follicle diameters at the beginning and end of the 72-hour culture period are depicted in Figure 2A. To determine the effects of alginate alone, follicles were encapsulated in calcium alginate, but grown in the complete absence of FSH as a negative control. A positive control comparison group consisted of follicles grown in the standard fashion and treated with FSH, but without encapsulation [7]. As expected, the negative control group of follicles encapsulated and grown in the absence of FSH, experienced minimal growth (diameter increase; 6.3%). The positive control group of unencapsulated follicles with FSH in the medium experienced a 35.4% increase in diameter, consistent with our previous studies [7]. When encapsulated follicles were grown in medium containing FSH, a 19.9% increase in follicle diameter over the 72-hour culture period was observed (p < 0.05 relative to the negative control). When FSH was included in the calcium alginate capsule but not the medium, a 13.5% increase in follicle diameter was observed. This was different from the negative control (p < 0.05), but not the previously described FSH in medium group (p > 0.05). However, when follicles were cultured with FSH included in both the medium and the bead, a 32.6% increase in follicle size was observed.

The size of positive control follicles and encapsulated follicles with FSH placed in the medium and the bead were significantly different than the other three treatment groups (Figure 2A, p < 0.05). Furthermore, there was no significant difference between the growth of unencapsulated FSH-treated cultures and the encapsulated follicles with FSH present in both the bead and the medium (positive control, 54.3 ± 10.1 μm; FSH in bead and media, 49.2 ± 6.8 μm; p > 0.05).

### Survival Analysis

The effect of encapsulation on follicle survival was evaluated by Kaplan Meier analysis of daily observation of cultured follicles. Survival plots for unencapsulated and encapsulated FSH-treated follicles are shown in Figure 2B. Mean intact survival time for encapsulated follicles, 6 days, was greater than the mean intact survival time for unencapsulated follicles, 4.5 days (p < 0.05).

### DNA Quantification confirms follicle growth

DNA content of individual follicles was consistent with measured follicle size. The unencapsulated follicles in FSH supplemented media (Positive Control) contained 50.7 ± 19.9 ng of DNA after 72 hours of culture. The encapsulated follicles with FSH present in the media and the bead (Experimental Group) contained 53.9 ± 15.9 ng of DNA, not significantly different from the positive control. Both FSH-treated groups contained significantly greater amounts of DNA than the encapsulated follicles cultured in the absence of FSH (Negative Control) which had only 27.8 ± 11.7 ng of DNA (p < 0.05).

### Connexin 43 expression in cultured follicles

**Figure 3:**
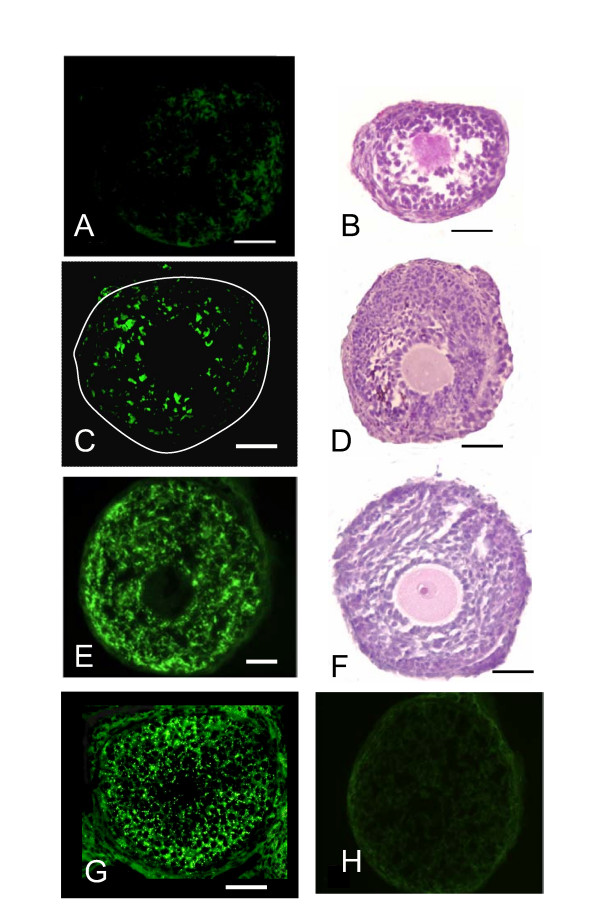
Connexin 43 expression and histology of representative cultured follicles. (A and B) Encapsulated follicle in the absence of FSH, (C and D) Unencapsulated follicle grown in FSH supplemented media, (E and F) Encapsulated follicle with FSH present in both the media and the bead, (G) Connexin staining in preantral follicle of an intact ovary section. (H) Negative control for the immunohistochemical staining, serial section from the same follicle as in C. A, C, E, G, H, represent connexin immunohistochemistry. B, D, and F are H&E stained follicle sections. In panel C, white line is location of basement membrane. Scale bars represents 50 μm in length.

As seen in Figure 3A, connexin expression (green stain) is very low in follicles in the absence of FSH. In the presence of FSH, connexin expression is readily apparent in the unencapsulated follicle (Figure 3C). However when the follicle is encapsulated and treated with optimal FSH (both in the alginate and in the medium), excellent expression of connexin 43 is seen throughout the cross section of the follicle (Figure 3E). This pattern is very similar to the follicle grown in vivo (Figure 3G). The negative control pictured (Figure 3H) is a serial section from the same follicle from 3E and demonstrates that there is minimal background staining in this system. Follicle sections stained with H&E are adjacent to the corresponding connexin photograph. In the absence of FSH (Figure 3B), the follicle remains intact but the granulosa cell layer is poorly organized and contains numerous pyknotic appearing cells. Figure 3D is of an FSH treated follicle that was not encapsulated. There is a thickened appearance of the outer granulosa cells though the cells around the oocyte appear fairly normal. This is consistent with the area that stains more heavily for connexin. Figure 3F depicts a typical follicle grown in the bead with optimal FSH. There is good morphology with a fairly uniform granulosa layer and a healthy appearing oocyte.

## Discussion

In this study, we have demonstrated that calcium alginate encapsulation can act as a scaffold to support three-dimensional relationships between the cells of a follicle in suspension culture. However we have also demonstrated that calcium alginate encapsulation can impede access of the follicle to FSH in the medium.

We have previously shown that FSH is a growth factor for preantral follicles in culture [19]. Other investigators have shown that the dosing and timing of follicle exposure to FSH has threshold limits for continued growth and survival of mouse follicles [21]. Our previous work has demonstrated that encapsulation slowed the rate of follicle growth [7]. There were two hypotheses considered to explain the decreased growth response to FSH. Either FSH diffusion into the alginate bead was significantly limited, or the bead itself physically hindered the growth of the follicle.

With FSH present only in the bead, a concentration gradient is created between the bead and the media. Naturally this gradient would drive the flux of FSH out of the calcium alginate capsule. For this case, because the molecular weight is less than 3 × 10^5^, the FSH will freely diffuse out of the bead until equilibrium is reached [17]. For a substrate with the molecular weight of FSH, equilibrium would be reached within 3 to 5 hours [11], resulting in a much lower concentration of FSH within the bead for the bulk of the 72 hour culture. As a result, follicular growth response was greatly diminished compared to the positive control (Figure 2).

Similarly, by adding FSH to only the media, a concentration gradient is established in the opposite direction, driving the flux of FSH from the media into the bead. However, due to the molecular weight of the hormone being greater than 2 × 10^4^, the diffusion into the capsule will be impeded [11]. The question is then, will the decreased diffusion rate into the calcium alginate bead be significant enough to cause the observed reduction in follicular growth (Figure 2).

To answer this, equal concentrations of FSH were placed in both the bead and the culture media. By eliminating the concentration gradients, FSH was not driven from the bead, leaving the concentration of FSH within the bead comparable to that present for the positive control. Therefore, because the addition of FSH to the alginate bead restored the follicular growth rate to that of the positive control, the likely cause of the diminution of follicle growth in our earlier studies [7] is a limitation of follicle access to FSH by the encapsulation process.

In order to confirm appropriate cell-cell interactions within the follicle, we next evaluated the expression of the gap junction protein connexin 43 in cultured follicles. Connexin 43 plays a key role in follicle development by promoting communication between granulosa cells via the exchange of small ions and molecules [22,23]. Connexin 43 deficient mice have severely impaired follicle development beyond the primary stage [24]. The expression and localization of connexin 43 is also highly FSH dependant [22]. The evaluation of connexin in our cultured follicles provides both a structural and a hormonal assessment of the effect of alginate on follicle development and FSH-stimulated differentiation.

In this study, it was found that the patterned expression of connexin 43 is not affected by calcium alginate encapsulation when the FSH is included in both the bead and the medium (Figure 3). Encapsulated preantral follicles cultured with FSH, maintain a well developed theca, a basement membrane, a normally configured granulosa compartment and a healthy appearing egg with a zona pellucida (Figure 3E and 3F). Interestingly, it was observed that the positive control lacked the punctuate staining at its periphery (Figure 3C), while the encapsulated follicle demonstrated consistent punctuate staining throughout the entire granulosa layer (Figure 3E) similar to in vivo preantral follicles (Figure 3G). This may suggest that encapsulating the follicle might protect it from possible deleterious shearing forces in the suspension culture system, since shear stress has been shown to affect connexin expression [25]. Further studies will be needed to more fully evaluate this phenomenon.

Although a variety of gels and scaffolds exist, calcium alginate has some desired advantages. Unlike collagen based gels [26,27], the follicle can be easily viewed through the calcium alginate capsule, allowing for daily observations to be made. Alginate is easily washed away with PBS, unlike collagen. The alginate also does not seem to interact with the follicles unless ECM or its components are included in the gel [28]. Matrigel has also been used as an imbedding medium for follicles. However matrigel is like serum in that it is derived from undefined biological sources and can have considerable content variation from lot to lot. In contrast, calcium alginate is a well-defined gel and fits with the goal of establishing a completely defined culture system that can be stably reproduced and still meet all the nutritional and structural needs of the developing follicle.

## Conclusion

These combined findings have demonstrated that microencapsulation can improve the support of three dimensional growth of preantral follicles; but requires the inclusion of FSH in the scaffold. Further work is necessary to determine if intact follicles can be maintained for longer periods in culture with this scaffolding approach. However, care must be taken to determine if the scaffolding material itself alters the microfollicular environment. Though follicle development is a prolonged and complex process, careful application of tissue engineering principles may facilitate the eventual development of a consistent, standardized *in vitro *process for follicle growth of large mammals that will be reliable enough for gamete production.

## List of Abbreviations

FSH: Follicle-Stimulating Hormone

DNA: Deoxyribonucleic Acid

MW: Molecular Weight

w/v: Weight per volume

IU: International Unit

ml: Milliliter

μm: Micrometer

mg: Milligram

L: Liter

Rpm: revolutions per minute

OCT: Optimal Cutting Temperature Compound

PBS: Phosphate Buffered Solution

Avidin DCS: Cell Sorting Grade Avidin D

ECM: extracellular matrix

## Authors' contributions

All authors participated in group discussions of the project conception and design and the analysis of data. MH and EAM drafted the manuscript, RK and AR edited and approved the final manuscript. All authors read and approved the final manuscript.
